# Increased Levels of Plasma Extracellular Heat-Shock Proteins 60 and 70 kDa Characterized Early-Onset Neonatal Sepsis

**DOI:** 10.3389/fped.2021.740274

**Published:** 2021-11-25

**Authors:** Arturo Alejandro Canul-Euan, Gibran Zúñiga-González, Janelly Estefania Palacios-Luna, Rolando Maida-Claros, Néstor Fabián Díaz, Patricia Saltigeral-Tigeral, Perla Karina García-May, Oscar Díaz-Ruiz, Héctor Flores-Herrera

**Affiliations:** ^1^Department of Inmunobioquímica, Instituto Nacional de Perinatología (INPer), Ciudad de México, Mexico; ^2^Department of Neonatología, Instituto Nacional de Perinatología (INPer), Mexico City, Mexico; ^3^Department of Fisiología y Desarrollo Celular, Instituto Nacional de Perinatología (INPer), Mexico City, Mexico; ^4^Instituto Nacional de Pediatría, Mexico City, Mexico; ^5^Servicio Recién Nacidos, Hospital Regional Lic. Adolfo López Mateos, Instituto de Seguridad y Servicios Sociales de los Trabajadores del Estado (ISSSTE), Mexico City, Mexico; ^6^Department of Pharmacology, Emory University School of Medicine, Atlanta, GA, United States

**Keywords:** early-neonatal sepsis, extracellular heat-shock protein, neonatal intensive care unit, tumor necrosis factor alpha, neonatal infection

## Abstract

**Background:** Extracellular heat-shock proteins (*e*Hsp) are highly conserved molecules that play an important role in inflammatory diseases and have been quantified in plasma from patients with infectious diseases, including sepsis. There is a constant search for dependable biochemical markers that, in combination with conventional methods, could deliver a prompt and reliable diagnosis of early-onset neonatal sepsis.

**Objective:** We sought to assess the level of *e*Hsp-27, *e*Hsp-60, *e*Hsp-70, and tumor necrosis factor-alpha (TNFα) in plasma of healthy neonates at term and infants with early-onset neonatal sepsis.

**Methods:** This study included 34 newborns that were classified as healthy neonates at term (blood samples from the umbilical cord, *n* = 23) or infants with early-onset neonatal sepsis (blood samples obtained from umbilical artery by standard sterile procedures before starting a systemic antibiotic intervention, *n* = 11). All blood samples were centrifuged, and the plasma recovered to determine *e*Hsp-27, *e*Hsp-60, *e*Hsp-70, and TNFα levels by ELISA.

**Results:** Our results indicate that the level of *e*Hsp-27 in healthy neonates at term was 0.045 ± 0.024 pg/ml. This value decreased 2.5-fold in infants with early-onset neonate sepsis (0.019 ± 0.006 pg/ml, *p* = 0.004). In contrast, the levels of *e*Hsp-60 and *e*Hsp-70 in healthy neonates at term were 13.69 ± 5.3 and 4.03 ± 2.6 pg/ml, respectively. These protein levels increased significantly 1.8- and 1.9-fold in the plasma of infants with early-onset neonatal sepsis (*p* ≤ 0.001). The level of TNFα in healthy neonates at term was 2.94 ± 0.46 pg/ml, with a 3.0-fold increase in infants with early-onset neonatal sepsis (8.96 ± 0.72 pm/ml, *p* ≤ 0.001). The sensitivity, specificity, positive predictive value (PPV), and negative predictive value (NPV) of *e*Hsp compared with that of C-reactive protein were 73.3, 60.0, 47.8, and 33.3%, respectively.

**Conclusion:** This study demonstrated a consistent increase of *e*Hsp-60 and *e*Hsp-70 in the plasma of infants diagnosed with early-onset neonatal sepsis. These proteins showed higher sensitivity and specificity than C-reactive protein and blood culture test.

## Introduction

Extracellular heat-shock proteins (*e*Hsp) are highly conserved molecules that regulate cellular homeostasis (1, 2), proliferation, and differentiation of the professional immune system cells and are modulated by temperature (3–5). The *e*Hsp have been classified in high molecular weight of 60, 70, 90, and 100 kDa and low molecular weight of 20 and 27 kDa (6, 7). When released into the extracellular space, *e*Hsp function as cell-to-cell mediators (8, 9). *e*Hsp-60 (HSPD1; heat shock protein family D member 1) and *e*Hsp-70 (HSPA1A; heat shock protein family A member 1A) can stimulate pro-inflammatory cytokines (10, 11), whereas *e*Hsp-27 (HSPB1; heat shock protein family B (small) member 1) has an important anti-inflammatory function (12–14). Their presence has also been shown and their levels quantified in serum and plasma of patients with severe trauma (15, 16), chronic obstructive pulmonary disease (17, 18), inflammatory processes induced by multiple sclerosis (19), and sepsis (20, 21). Therefore, *e*Hsp have been used as sensible indicators of the physiological status during the onset and resolution of different human pathological conditions (19, 22, 23).

Neonatal sepsis is a common and serious disease that affects a large number of newborns around the world. Although its incidence is low (one to eight cases for every 1,000 live births) (24), the risk of morbidity and mortality is high, affecting 15–50% of reported cases (24, 25). In developed countries, the estimated prevalence is 2–8% (24). The Department of Neonatal Intensive Care of the National Institute of Perinatology “Isidro Espinosa de los Reyes” (INPer*IER*) in Mexico City has reported an incidence of 2.3% in the total number of births attended during a 5-year period (26).

The clinical diagnosis of early-onset neonatal sepsis poses challenges due to the subtlety of signs and symptoms, which are often concealed with other transient medical conditions such as hypothermia, delayed transition from fetal to neonatal life, tachypnea, and metabolic alterations (25). The clinical identification and diagnosis of neonatal sepsis is confirmed by blood culture (27, 28), and the assessment of acute phase reactants includes C-reactive protein (CRP) (29, 30), procalcitonin (30, 31), presepsin (32, 33), and inflammatory mediators such as interleukin-6 (IL-6) and tumor necrosis factor-alpha (TNFα) (34, 35). It has been shown that, when two or more tests are combined, the accuracy of a prompt diagnosis of early-onset neonatal sepsis increases (36, 37). At INPer*IER*, some of these acute phase reactants or biological markers have not provided the accuracy and sensitive to support clinical data in the diagnosis of early-onset neonatal sepsis. Interestingly, several reports have shown that *e*Hsp are reliable and practical biomarkers to identify sepsis in children (21, 38). In this study, we set out to quantify the *e*Hsp and TNFα in plasma of healthy neonates at term and infants with early-neonatal sepsis.

## Materials and Methods

### Ethics Statements

This study was reviewed and approved by the National Institute of Perinatology Ethics and Research Committees (registration number 212250-3210101). All patients were informed about the purpose of the study and a maternal informed consent obtained in all cases.

### Study Design and Patients

From July 2018 to June 2019, a cross-sectional study was carried out in the Neonatal Intermediate Therapy Unit for the newborn. A total of 34 newborns were included. The neonates were divided into two groups: (1) healthy neonates at term (blood samples obtained at birth from the umbilical cord, *n* = 23) and (2) neonates with visible signs of early-onset neonatal sepsis (blood samples obtained from umbilical artery by standard sterile procedures before starting a systemic antibiotic intervention; *n* = 11).

### Clinical Definitions and Inclusion Criteria

Healthy neonates consisted of neonates at term, gestational age ≥37 weeks, delivery without obstetric complications of labor and/or signs of maternal sepsis. Neonates with early-onset neonatal sepsis consisted of infants with visible signs and symptoms (feeding intolerance, lethargic or tachypnic, poor perfusion, seizures, respiratory distress, bradycardia, abdominal distension, or vomiting) normally associated with suspected sepsis as defined according to the guidelines for the management of newborns with suspected sepsis (39–41).

Some of the maternal patients presented the following conditions: preterm rupture of fetal membranes (pPROM), which was diagnosed by discharge of amniotic fluid through the vaginal canal or by a positive nitrazine test (42); clinical chorioamnionitis (CAM) was diagnosed by the presence of fever (>38°C), accompanied by two or more of the following signs: tachycardia (heart rate >100 beats per min), uterine pain or tenderness, fetid or purulent amniotic fluid, leukocytosis >15,000/mm^3^, CRP (>2 mg/dl), and fetal tachycardia (heart rate >160 beats per minute) (43–45).

### Exclusion Criteria

Neonatal sepsis cases were excluded from the study when (1) the amount of blood collected for the quantification of *e*Hsp and TNFα was insufficient (plasma < 1,200) and (2) antibiotic treatments started prior to blood collection.

### Blood Sample Collection

Two milliliters of blood were obtained by trained medical staff. The blood samples were collected in K_2_-EDTA vacutainer tubes (Becton-Dickinson, NJ, USA) and centrifuged at 329 g (Beckman, GS-6R Centrifuge) for 5 min. The plasma was recovered in Eppendorf tubes and stored at −80°C until quantification of the *e*Hsp-27, *e*Hsp-60, *e*Hsp-70, and TNFα by enzyme-linked immunosorbent assay (ELISA).

### Biochemical Assays

Commercial ELISA kits were used to quantify the levels of *e*Hsp-27 (DYC-1580, R&D Systems, Minneapolis, MN, USA), *e*Hsp-60 (DYC1800-2, R&D Systems), *e*Hsp-70 (DYC1663-2, R&D System), and TNFα (DY210, R&D System). The plasma used for the quantified was not diluted. Standard curves were calculated from 31.3 to 2,000 ng/ml, 1.25 to 80 ng/ml, 312.5 to 20,000 pg/ml, and 15.0 to 960 pg/ml, respectively, according to the manufacturer's instructions and the protocol previously reported by our research group (46). The following sensitivity values for each protein were calculated 50, 0.70, 150.0, and 5.0 pg/ml, respectively. The CRP levels were determined a few hours after the blood was obtaining. CRP was measured by nephelometry using a MININEPH PLUS System (Birmingham, UK) and with commercial kit (ZK044.L.R, Birmingham, UK) according to the manufacturer's instructions. CRP assay has a detection range of 6 to 1,232 mg/L and Inter- and Intra-Assay Coefficients of Variability <4% both at low and high concentration. CRP was processed at INPer*IER* core facility.

### Microbiological Analysis

Bacterial culture analysis and identification test for aerobic and anaerobic microorganisms were performed at the Department of Infectología e Inmunología at the INPer*IER*. The procedure was performed with the Bact/Alert 3D (Dirham, BioMerieux, NC, USA) as previously reported by Thorpe et al. (47).

### Statistical Analysis

Chi-square test was used to compare maternal and neonatal clinical data. *e*Hsp and TNFα levels in healthy neonates at term and infants with early-onset neonatal sepsis were analyzed using one-way ANOVA and significant difference between groups were determined by the Tukey's test. All assays were independently replicated at least three times, and the data are presented as mean ± SEM. Statistical analyses were carried out using SigmaStat software (version 3.0). A significant difference was accepted at *p* ≤ 0.05.

## Results

### Demographic Data of the Study Population

Table 1 shows the demographic and clinical characteristics of maternal and neonatal patients included in this study. In maternal patients, no significant difference between groups was detected in age (*p* = 0.423), body mass index (*p* = 0.927), and number of neonates delivered alive during the first three pregnancies, but a significant difference during the fourth pregnancy (9.0 vs. 0.0%, *p* = 0.013) was found.

**Table 1:** Demographic and clinical characteristics of maternal and neonatal patients.

**Characteristics**	**Healthy neonates at term (*n* = 23)**	**Neonates with early-onset sepsis (*n* = 11)**	***p*-value**
Maternal conditions			
Age, year	28.3 ± 7.5	26.2 ± 7.1	0.423
Body mass index, kg/cm^2^	25.7 ± 6.5	26.3 ± 8.2	0.927
Number of previous pregnancies alive			
0 *n* (%)	11 (48)	5 (46)	0.887
1 *n* (%)	4 (17)	2 (18)	0.851
2 *n* (%)	5 (22)	4 (36)	0.207
3 *n* (%)	1 (4)	0 (0)	0.214
4 *n* (%)	2 (9)	0 (0)	0.013
CAM, *n* (%)	0 (0)	4 (36)	0.013
pPROM, *n* (%)	0 (0)	3 (27)	0.001
CAM + pPROM, *n* (%)	0 (0)	1 (9)	0.013
PE, *n* (%)	0 (0)	3 (27)	0.001
Severe PE, *n* (%)	0 (0)	2 (18)	0.001
Fever >38°C, *n* (%)	0 (0)	0 (0)	
Histological inflammation			
Fetal membranes, *n* (%)	0 (0)	1 (9)	0.013
Umbilical, *n* (%)	0 (0)	0 (0)	1.0
Placental, *n* (%)	0 (0)	0 (0)	1.0
Neonatal conditions			
Gender			
Male, *n* (%)	10 (43)	5 (45)	0.886
Female, *n* (%)	13 (56)	6 (54)	
Gestational age (weeks)	38.6 ± 1.1	33.0 ± 3.3	0.001
Birth weight (g)	2,970.5 ± 441.0	1,380 ± 804.8	0.006
Irritability	0 (0)	0 (0)	
APGAR at 5 min <8, *n* (%)	0 (0)	9 (82)	0.001
Fever >38°C, *n* (%)	0 (0)	0 (0)	

*CAM, Chorioamnionitis; pPROM, preterm-prelabor rupture of membranes; PE, pre-eclampsia*.

Maternal patients who delivered infants with early-onset neonatal sepsis developed clinical CAM in 36.0% of cases, whereas pPROM occurred in 27.0%. Only 9.0% presented CAM and pPROM simultaneously. Furthermore, 27.0% of maternal patients showed clinical preeclampsia (PE) and 18.0% of them had severe PE (Table 1) and 8.3% of newborns had intrauterine growth restriction (Sample 5, Table 2).

**Table 2:** Clinical diagnosis of early-onset neonatal sepsis with bacterial detections.

**Neonatal blood sample**	**Sex**	**Gestational age** **(w)**	**Birth weight** **(g)**	**Bacteria detected**	**Clinical diagnosis and maternal condition**
1	F	32	3,302	ND	CAM
2	F	33	1,170	*E. coli*	CAM
3	F	31	1,060	ND	7 days of pPROM
4	M	34	2,485	ND	8 days of pPROM, without clinical data of CAM
5	F	30	1,300	ND	Severe PE and RCIU
6	M	35	1,245	*S. epidermidis*	Without clinical pathological data
7	M	28	1,085	ND	Severe PE
8	M	37	2,700	*S. dysgalactiae*	PE
9	F	39	2,640	ND	Without clinical pathological data
10	F	30	1,380	ND	CAM
11	M	33	2,094	ND	2 days of pPROM

*CAM, chorioamnionitis; pPROM, preterm-prelabor rupture of membranes; PE, pre-eclampsia; ND, not detected*.

In neonatal patients, we found significant differences between groups. Infants with early-onset neonate sepsis showed 1.2-fold decrease in gestational age compared with healthy neonates at term (38.6 ± 1.1 weeks; *p* ≤ 0.001); 2.15-fold decreased in body weight at birth (2,970.5 ± 441.0 vs. 1,380 ± 804.8 kg; *p* = 0.006), and 82% of infants with early-onset neonatal sepsis showed APGAR < 8 at 5 min. No gender-based difference was found (Table 1).

### Microbiological Analysis

Table 2 shows the bacteria detected in blood culture, maternal diagnosis, and evidence of sepsis. Blood culture was positive in 27.3% of samples taken from infants with early-onset neonatal sepsis (three of 11 cases). The bacteria identified in these samples were *E. coli* (1 case, S2), *E. epidermidis* (1 case, S6), and *S. dysgalactiae* (1 case, S8). Blood cultures of samples taken from healthy neonates at term were negative (Table 2).

### Extracellular Heat-Shock Proteins and Inflammatory Cytokine in Plasma

**Figure 1:**
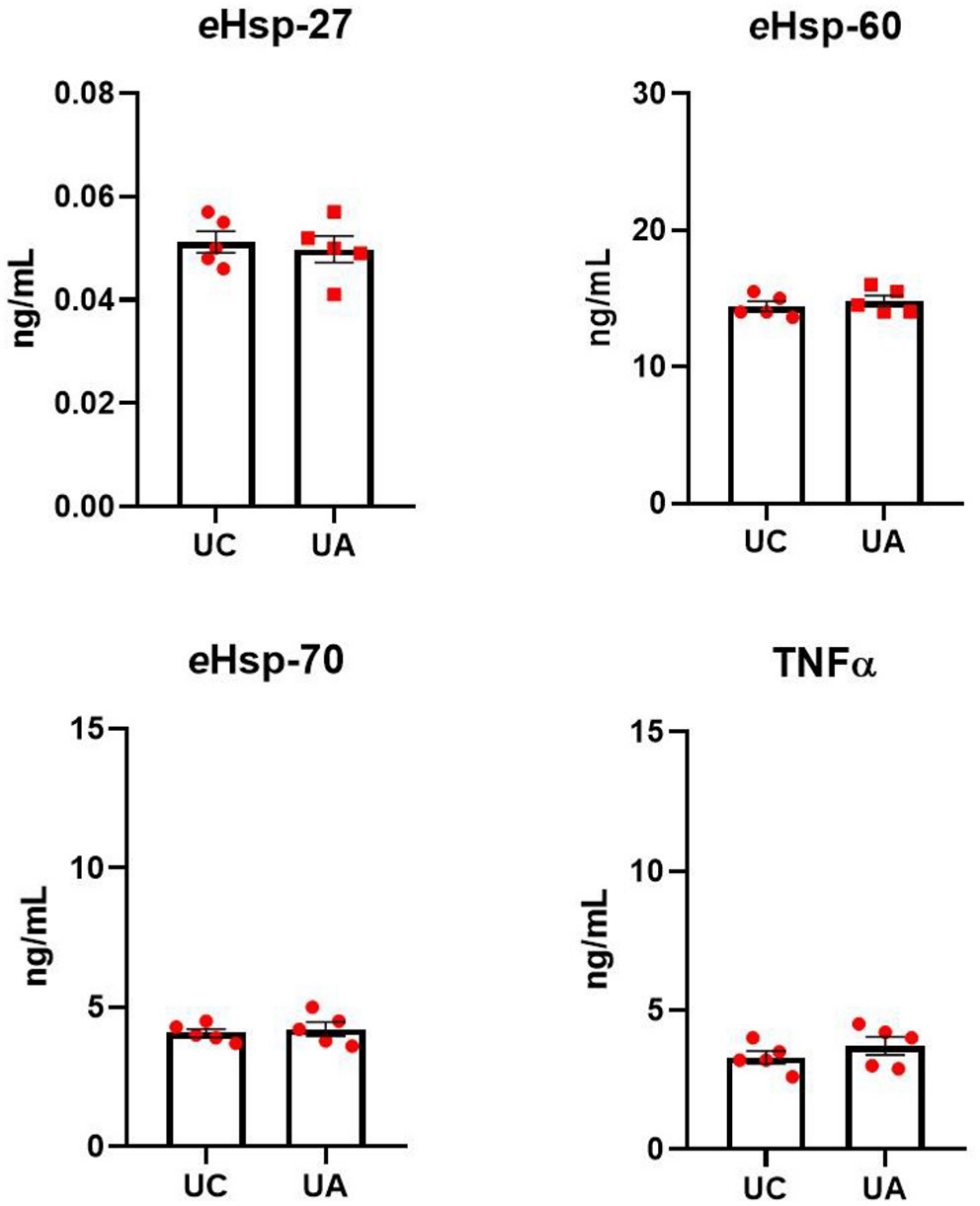
Quantification of *e*Hsp-27, *e*Hsp-60, *e*Hsp-70, and TNFα in plasma from blood samples collected from the umbilical cord (UC) and the umbilical artery (UA) in healthy neonates at term (*n* = 5). Concentration was expressed as ng/ml. Data represent the mean ± SEM.

To assess whether blood from different sampling sites does not affect the quantification of eHsp and TNFα, blood samples from both the umbilical cord and the umbilical artery from five different healthy neonates at term were collected. All samples were assessed for eHsp and TNFα. No differences were found (Figure 1) between sampling sites for *e*Hsp-27 (0.051 ± 0.004 vs. 0.04 ± 0.005, *p* = 0.6851), *e*Hsp-60 (14.4 ± 0.79 vs. 15.1 ± 1.02, *p* = 0.1958), *e*Hsp-70 (4.08 ± 0.31 vs. 4.22 ± 0.55, *p* = 0.6396), and TNFα (3.3 ± 0.50 vs. 3.7 ± 0.72, *p* = 0.3207).

**Figure 2:**
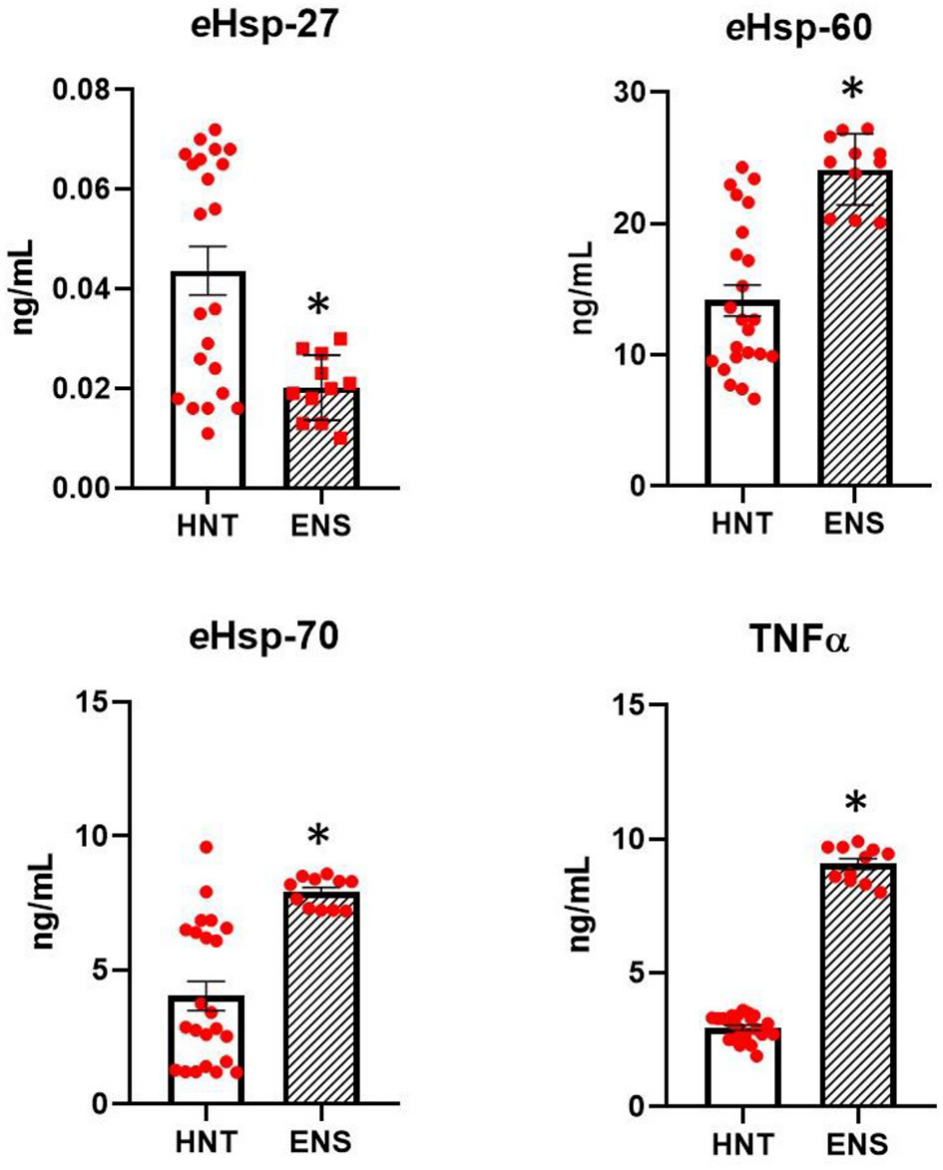
Comparison in the secretion of *e*Hsp-27, *e*Hsp-60, *e*Hsp-70, and TNFα between healthy neonates at term (HNT, *n* = 23) and neonates with early-onset neonatal sepsis (ENS, *n* = 11). Concentration was expressed as ng/ml. Data represent the mean ± SEM. Statistical difference was observed between both groups *p ≤ 0.001.

Figure 2 shows the levels of *e*Hsp-27, *e*Hsp-60, *e*Hsp-70, and TNFα in the plasma of healthy neonates at term and infants with early-onset neonatal sepsis. The levels of *e*Hsp-27 decreased 2.2-fold in infants with early-onset neonatal sepsis compared with healthy neonates at term (0.045 ± 0.02 vs. 0.019 ± 0.006 pg/ml, *p* = 0.008; Figure 2). In contrast, the levels of *e*Hsp-60, *e*Hsp-70, and TNFα increased significantly in all infants with early-onset neonatal sepsis compared with healthy neonates at term 1.6-fold (14.15 ± 5.7 vs. 24.7 ± 3.0 pg/ml, *p* ≤ 0.001), 2.0-fold (4.03 ± 2.6 vs. 7.9 ± 0.62 pg/ml, *p* ≤ 0.001), and 3.0-fold (2.94 ± 0.46 vs. 8.96 ± 0.72 pg/ml, *p* ≤ 0.001), respectively (Figure 2).

Table 3 shows the relevance of assessing *e*Hsp levels as part of routine clinical laboratory tests for early-onset neonatal sepsis. The sensitivity and specificity of *e*Hsp compared with that in CRP test of 11 samples from infants with early-onset neonatal sepsis were 73.3 and 63.2%, respectively. In addition, positive predictive value (PPV) and negative predictive value (NPV) values were 47.8 and 36.4%, respectively. Finally, the sensitivity and specificity of *e*Hsp compared with that of blood culture were 73.3 and 60.0%, whereas PPV and NPV values were 47.8 and 33.3%, respectively.

**Table 3:** Comparison between clinical laboratory and biochemical test in early-onset neonatal sepsis.

**Neonatal blood sample**	**Clinical laboratory test**		**Biochemical test**		
	**CRP (ng/ml)**	**BC**	***e*Hsp-60 (ng/ml)**	***e*Hsp-70 (ng/ml)**	**TNFα (ng/ml)**
1	ND	–	20.2	7.3	8.3
2	57	+	20.3	7.2	7.8
3	ND	–	25.3	7.7	8.0
4	ND	–	27.1	8.2	8.45
5	6	–	26.6	7.2	9.7
6	ND	–	24.7	8.4	9.6
7	8	+	24.7	8.6	9.4
8	56	+	25.3	8.5	9.3
9	ND	–	27.2	7.2	9.7
10	ND	–	23.8	6.9	8.6
11	ND	–	17.9	8.3	9.9

*CRP, C-reactive protein; BC, blood culture; eHsp, extracellular heat-shock protein; TNFα, tumor necrosis factor-alpha; ND, not detected*.

## Discusion

*e*Hsp have traditionally been considered as intracellular molecules involved in cellular protections (48, 49). However, in recent years, they have been reported as molecules related to different components of the immune response (5, 50). It has been shown that *e*Hsp-60 and *e*Hsp-70 proteins are associated with the inflammatory response (21, 38, 51) and are increased in the plasma of children with septic shock (52, 53). Notwithstanding, the role of *e*Hsp in the plasma of infants with clinical evidence of early-onset neonatal sepsis is poorly unknown.

The main findings of this study are as follows: (1) there is an upregulation of *e*Hsp-60 and *e*Hsp-70 in plasma of patients with early-onset neonatal sepsis, in parallel with an increment of TNFα level that has been previously reported as an early-onset neonatal sepsis biomarker; (2) the downregulation of *e*Hsp-27 in plasma of patients with early-onset neonatal sepsis indicates an inverse relationship with the levels of *e*Hsp-60 and *e*Hsp-70; (3) the high levels of *e*Hsp-60 and *e*Hsp-70 in plasma were consistently detected in neonates with visible signs and symptoms of sepsis even in cases with an undetectable level of CRP and bacteria in blood cultures (Table 3); (4) *e*Hsp-60 and *e*Hsp-70 tests showed higher sensitivity and specificity compared with CRP and blood culture tests.

Studies by Wheeler et al. (54) and He et al. (55) in children with severe sepsis have shown a significant increase in the levels of *e*Hsp-70, TNFα, IL-1β, IL-6, IL-8, IL-13, IL-27, macrophage inflammatory protein-1α, and matrix metalloproteinase-8 (MMP-8) in blood and plasma (54, 56). Studying the systemic inflammatory response syndrome in children, Fitrolaki et al. (51) demonstrated increased levels Hsp-72, Hsp-90, IL-8, IL-6, and TNFα in patients diagnosed with sepsis and considered these as biomarkers associated with fatal outcome in these patients (51, 57). In this investigation, we showed that infants with early-onset neonatal sepsis presented increased levels of *e*Hsp-60 and *e*Hsp-70 that are correlated with an increment in TNFα (Table 3), supporting previous evidence reported by our group and replicating previous reports (46, 51).

A positive blood culture is considered the gold standard for the diagnosis and identification of many clinical infections (27, 58). However, it has a low sensitivity and specificity when used to diagnose neonatal sepsis (59). Recently, it has been shown that neonatal sepsis produced by Group B *Streptococcus, Escherichia coli, Enterococcus faecalis, Staphylococcus epidermidis, Streptococcus pneumonia, Acinetobacter baumannii*, and *Neisseria meningitidis* is associated with increased levels of *e*Hsp-70, *e*Hsp-90, and TNFα in blood and plasma (51, 55). Our findings provide new evidence and support previous results showing that infants with early-onset neonatal sepsis with positive blood culture for *E. coli, S. epidermidis*, and *S. dysgalactiae* also display marked increase levels of *e*Hsp-60, *e*Hsp-70, and TNFα in plasma.

Using experimental models of infection Campisi et al. (60) showed that *E. coli* induces a dose-dependent expression of *e*Hsp-72, which is mediated by Toll-like receptor (TLR) by recognizing different structural components of bacteria (60–62). The secretion of *e*Hsp-72 has been associated with increased levels of other biomarkers, such as nitric oxide, TNFα, IL-1β, and IL-6 (60).

**Figure 3:**
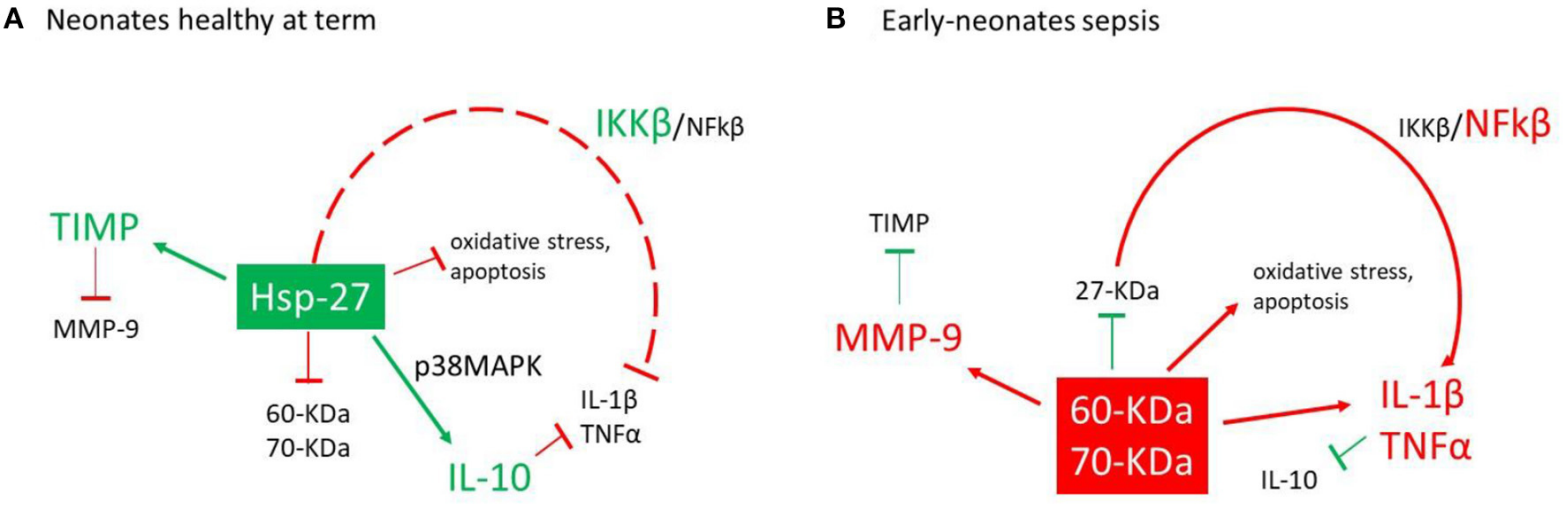
Action model for the activation of *e*Hsp and proinflammatory cytokines. **(A)** In healthy neonates at term, *e*Hsp-27 modulates the expression of the protein inhibitor (Ikβ) reducing the activity of the nuclear factor-kappa β (NFkβ) pathway (17, 31), modulating the inflammatory response (17, 47), oxidative stress (39), and action of matrix metalloproteinases-9 (MMP-9) (49, 52). **(B)** During the infections process, the inflammatory response is activated which reduces the expression of *e*Hsp-27, increased the expression of *e*Hsp-60, *e*Hsp-70, and NFkβ and finally increases the secretion of IL-1β, TNFα, and MMP-9 (48, 51, 53).

Figure 3 shows a proposed model for the differential actions of the anti-inflammatory (*e*Hsp-27) and proinflammatory (*e*Hsp-60 and *e*Hsp-70) response (5, 9). In healthy patients, *e*Hsp-27 is the mainly expressed *e*Hsp, and it is related to protein inhibitory β, a negative regulator of the classical nuclear transcription factor-kappa β (NFkβ) pathway (5, 50), which reduce the production of molecules associated with oxidative stress (63), apoptosis (64, 65), IL-1β, TNFα (12, 21), and collagenolytic action of MMP-9 (66, 67) (Figure 3A). During infection, the inflammatory response is activated, reducing the expression of *e*Hsp-27, increasing the activity of NFkβ and enhancing the levels of *e*Hsp-60 and *e*Hsp-70. This chain of events upregulates IL-1β, TNFα, and MMP-9 (68–70). In sepsis, this activation is mediated by TLR-4 (61, 62) (Figure 3B). Interestingly, our data demonstrate a significant imbalance between a decrease of anti-inflammatory *e*Hsp-27 and an increase of pro-inflammatory *e*Hsp-60 and *e*Hsp-70 in infants with early-onset neonatal sepsis.

Clinical guidelines in cases of possible early-onset neonatal sepsis require both CRP assessment and positive blood culture. CRP is produced by the liver and is increased in response to early-onset neonatal sepsis (71). However, several studies have often show inconsistency in the assessment of CRP, possibly due to the gestational age and volume required for testing (30). Nevertheless, the sensibility of these tests increases when additional markers are assessed (72).

The most remarkable finding of our study is that the levels of *e*Hsp-60 and *e*Hsp-70 were consistently increased in all infants with early-onset neonate sepsis (Table 3), whereas blood culture and CRP tests when combined were able to detect 27.27% of the positive cases. The sensitivity, specificity, PPV, and NPV for *e*Hsp with regard to the CRP test was 73.3, 63.2, 47.8, and 36.4%, respectively.

## Conclusion

Our study highlights that *e*Hsp-60 and *e*Hsp-70 measured in the plasma of infants could be used as a reliable biomarker of early-onset neonatal sepsis, because the levels of these proteins are consistently elevated and show high sensitivity, specificity, PPV, and NPV. These results provide a strong indication that the assessment of these proteins, together with conventional tests such as CRP and blood culture, can provide a highly sensitive and accurate diagnostic tool to confirm diagnosis of early-onset neonatal sepsis.

## Data Availability Statement

The raw data supporting the conclusions of this article will be made available by the authors, without undue reservation.

## Ethics Statement

The studies involving human participants were reviewed and approved by Comiteé de Ética en Investigación. Instituto Nacional de Perinatología. The patients/participants provided their written informed consent to participate in this study.

## Author Contributions

AC-E, GZ-G, and RM-C obtained blood samples from neonates with and without evidence of early-neonatal sepsis. AC-E and GZ-G provided clinical data on neonates. AC-E, GZ-G, JP-L, and HF-H performed the quantification of *e*Hsp. JP-L performed the quantification of TNFα. PS-T, PG-M, and HF-H conceived and designed the study. AC-E, GZ-G, ND, PS-T, OD-R, and HF-H analyzed the data and interpreted the results. PS-T, PG-M, OD-R, and HF-H wrote the manuscript. All other authors gave approval for the final version of manuscript.

## Funding

This research was supported by a grant (number 212250-3210101 assigned to HF-H) from the National Institute of Perinatology, Mexico City. This paper is part of the experimental work of AC-E required for obtaining the Master in Science degree (number 516222901) from Programa de Ciencias Medicas, Odontológicas y de la Salud, Universidad Nacional Autónoma de México (UNAM). We thank CONACyT for supporting his studies.

## Conflict of Interest

The authors declare that the research was conducted in the absence of any commercial or financial relationships that could be construed as a potential conflict of interest.

## Publisher's Note

All claims expressed in this article are solely those of the authors and do not necessarily represent those of their affiliated organizations, or those of the publisher, the editors and the reviewers. Any product that may be evaluated in this article, or claim that may be made by its manufacturer, is not guaranteed or endorsed by the publisher.
